# Efficacy of goserelin plus anastrozole in premenopausal women with advanced or recurrent breast cancer refractory to an LH-RH analogue with tamoxifen: Results of the JMTO BC08-01 phase II trial

**DOI:** 10.3892/or.2013.2312

**Published:** 2013-02-27

**Authors:** REIKI NISHIMURA, KEISEI ANAN, YUTAKA YAMAMOTO, KENJI HIGAKI, MAKI TANAKA, KENJI SHIBUTA, YASUAKI SAGARA, SHINJI OHNO, SHIGERU TSUYUKI, TAKAHIRO MASE, SATOSHI TERAMUKAI

**Affiliations:** 1 Department of Breast and Endocrine Surgery, Kumamoto City Hospital, Kumamoto 862-8505, Japan; 2 Department of Surgery, Kitakyushu Municipal Medical Center, Kitakyushu 802-0077, Japan; 3 Department of Breast and Endocrine Surgery, Kumamoto University Hospital, Kumamoto 860-8556, Japan; 4 Department of Breast Surgery, Hiroshima City Hospital, Hiroshima 730-8518, Japan; 5 Department of Surgery, Social Insurance Kurume Daiichi Hospital, Kurume 830-0013, Japan; 6 Department of Breast Surgery, Ueo Breast Surgical Hospital, Oita 870-0854, Japan; 7 Department of Breast/Thyroid Gland, Hakuaikai Medical Corporation, Sagara Hospital, Kagoshima 892-0833, Japan; 8 Division of Breast Oncology, National Kyushu Cancer Center, Fukuoka 811-1395, Japan; 9 Department of Breast and General Surgery, Osaka Red-Cross Hospital, Osaka 543-8555, Japan; 10 Department of Breast and Endocrine Surgery, Ichinomiya Municipal Hospital, Ichinomiya 491-8558, Japan; 11 Department of Clinical Trial Design and Management Translational Research Center, Kyoto University Hospital, Kyoto 606-8507, Japan

**Keywords:** aromatase inhibitor, breast cancer, luteinizing hormone-releasing hormone analogue, premenopausal patient, tamoxifen

## Abstract

The aim of the present study was to assess the efficacy and tolerability of a luteinizing hormone-releasing hormone (LH-RH) analogue plus an aromatase inhibitor following failure to respond to standard LH-RH analogue plus tamoxifen (TAM) in premenopausal patients. Premenopausal women with estrogen receptor (ER)-positive and/or progesterone-receptor positive, advanced or recurrent breast cancer refractory to an LH-RH analogue plus TAM received goserelin (GOS) in conjunction with anastrozole (ANA). The primary endpoint was the objective response rate (ORR). Secondary endpoints included progression-free survival (PFS), overall survival (OS), clinical benefit rate (CBR) and safety. Between September 2008 and November 2010, 37 patients were enrolled. Thirty-five patients (94.6%) had ER-positive tumors, and 36 (97.3%) had human epidermal growth factor receptor (HER) 2-negative tumors. Thirty-six (97.3%) had measurable lesions and 1 (2.7%) had only bone metastasis. The ORR was 18.9% [95% confidence interval (CI), 8.0–35.2%], the CBR was 62.2% (95% CI, 44.8–77.5%) and the median PFS was 7.3 months. Eight patients had adverse drug reactions but none resulted in discontinuation of treatment. GOS plus ANA is a safe effective treatment for premenopausal women with hormone receptor-positive, recurrent or advanced breast cancer. The treatment may become viable treatment in the future, particularly when TAM is ineffective or contraindicated. Further studies and discussion are warranted.

## Introduction

Approximately 70% of all cases of breast cancer are hormone receptor-positive. Endocrine therapy is generally used for adjuvant treatment and the management of recurrence in hormone-sensitive breast cancer. Ovarian suppression induced surgically or with a luteinizing-hormone-releasing hormone (LH-RH) analogue as a postoperative adjuvant therapy can prevent recurrence and prolong survival in premenopausal women with breast cancer. The effectiveness of these treatments is comparable to that of chemotherapy (1,2). In premenopausal women, estrogen is synthesized primarily by the ovaries, and high estrogen concentrations are maintained in the blood. After menopause, the decline in ovarian function is accompanied by a significant decrease in estrogen concentrations in the blood, although levels remain high enough to stimulate the proliferation of breast cancer cells. Estrogen in postmenopausal patients is largely produced in peripheral adipose tissue and in cancer cells, and the peripheral aromatase is not under gonadotropin regulation (3). Therefore, aromatase inhibitors are used as standard treatment in postmenopausal women with breast cancer following the cessation of ovarian function. Particularly in patients with recurrent or metastatic breast cancer, the major treatment objectives are to maintain or improve the quality of life (QOL) and to prolong survival. Treatment should therefore be initiated with endocrine therapy.

Endocrine therapy basically involves sequential administration of single agents. However, the combined use of an LH-RH analogue and tamoxifen (TAM) is superior to monotherapy (4) and is, therefore, the treatment of choice for premenopausal women with advanced or recurrent breast cancer. However, when the disease is resistant to combination therapy involving LH-RH analogue and TAM, alternative regimens for endocrine therapy are currently unavailable, with the exception of synthetic progesterone agents (medroxyprogesterone acetate). A number of patients must therefore receive chemotherapy. Consequently, the National Comprehensive Cancer Network (NCCN) Clinical Practice Guidelines recommend that premenopausal women with advanced or recurrent breast cancer undergo ovarian ablation or suppression and then receive treatment similar to that recommended for postmenopausal women. The above mentioned guidelines recommend that premenopausal breast cancer patients undergo a combination treatment that includes an LH-RH analogue and an aromatase inhibitor. However, few studies support this treatment regime for premenopausal patients. Forward *et al*(5) studied goserelin (GOS) plus anastrozole (ANA) as a second-line endocrine therapy in 16 premenopausal women with advanced breast cancer who had previously received an LH-RH analogue plus TAM. After 6 months of treatment, 1 patient had partial response (PR), 9 had stable disease (SD) and 2 had a biochemical response. The clinical benefit rate was 75%. Serum estradiol levels were measured during treatment. Introduction of GOS and TAM reduced mean estradiol levels by approximately 89%. Substitution of TAM with ANA further decreased estradiol levels by 76%. This represents a marked decrease compared with the level during treatment using GOS and TAM.

These results suggest that combination therapy with an LH-RH analogue and an aromatase inhibitor is a viable treatment option for premenopausal women with breast cancer. To confirm this hypothesis, we studied the response rate to an LH-RH analogue plus ANA in women who failed to respond to an LH-RH analogue plus TAM. Progression-free survival (PFS), overall survival (OS), clinical benefit rate (CBR) and safety were also assessed.

## Patients and methods

### Study design

This open-label, single-arm, multi-center, phase II study (registration no. UMIN000001217) was conducted to assess the efficacy and safety profile of an LH-RH analogue and an aromatase inhibitor combination therapy in patients with TAM-refractory, ER-positive, premenopausal metastatic breast cancer in Japan between September 2008 and February 2012. The following treatment was initiated within 4 weeks after enrollment. Anastrozole (Arimidex) 1-mg tablets were administered orally once daily. A 3.6-mg depot of GOS acetate (Zoladex) was injected subcutaneously into the lower abdomen once every 4 weeks (28 days). Treatment was continued until the development of progressive disease (PD) or unacceptable adverse events.

This study was conducted in accordance with the Declaration of Helsinki, and the Ethical Guidelines for Clinical Studies, July 30, 2003 (Amended December 28, 2004) by the Ministry of Health, Labor and Welfare, Japan. This protocol was approved by JMTO (The Japan-Multinational Trial Organization) Ethics Committee in February 2008 and was also approved by the Ethics Committee of each institution. The local assessment [complete response (CR), PR or prolonged SD of ≥24 weeks] was confirmed independently by two radiologists.

### Eligible patients

Eligible patients had to meet all of the following inclusion criteria at study entry: premenopausal women 20–55 years of age (at enrollment); a confirmed diagnosis of metastatic or recurrent breast cancer; measurable lesions [according to Response Evaluation Criteria in Solid Tumors (RECIST)] or assessable bone lesions; refractoriness to previous treatment with an LH-RH analogue plus TAM; compliance with one of the following four conditions: i) recurrence while receiving postoperative therapy with an LH-RH analogue plus TAM; ii) recurrence within 1 year after the completion of at least 2 years of postoperative treatment with an LH-RH analogue plus TAM; iii) recurrence while receiving postoperative treatment with TAM alone after at least 2 years of treatment with an LH-RH analogue plus TAM or recurrence within 1 year after the completion of treatment with TAM, or iv) progressive disease while receiving combination therapy with an LH-RH analogue plus TAM for the management of advanced or recurrent breast cancer; estrogen receptor (ER)- and/or progesterone receptor (PgR)-positive breast cancer (positivity rate ≥10% on immunohistochemical analysis), an Eastern Cooperative Oncology Group performance status of 0 or 1; in patients who were receiving bisphosphonates, measurable lesions in sites other than the bone able to be followed up for antitumor response; with no serious complications; and written informed consent to participate in the study, received directly from the patient.

Patients were excluded from the study if they met any of the following criteria: i) a history of allergy to the study drug or concurrently used drugs; ii) treatment with other antitumor agents after prior therapy (LH-RH analogue plus TAM or LH-RH analogue plus TAM→TAM); iii) continuous treatment with systemic corticosteroids (orally or intravenously); iv) advanced cancer in other organs <5 years after treatment; v) a history of thrombosis, such as deep vein thrombosis or cerebral infarction; vi) a history of serious cardiac disease, such as myocardial infarction, valvular disease, or heart failure; vii) hormone-replacement therapy for climacteric symptoms received for ≤4 weeks at the time of enrollment; viii) women who were pregnant, breast feeding, or possibly (planning to be) pregnant; ix) treatment with antineoplastic agents other than an LH-RH analogue plus ANA, bisphosphonates, or radiotherapy of target lesions scheduled to be received after the start of the study; and x) patients considered unsuitable for the study by the investigator.

### Study variables

The variables investigated included age, body-mass index, tumor diameter of the primary lesion, lymph-node metastasis, ER, PgR, human epidermal growth factor receptor (HER) 2 status, sites of metastasis or recurrence, performance status at enrollment (according to the Eastern Cooperative Oncology Group), the presence or absence of postoperative radiotherapy, and the presence or absence of chemotherapy. Immunohistochemical staining was used to evaluate ER, PgR and HER2. ER and PgR were judged to be positive if the percentage of positive cells was ≥10%. HER2-positivity was defined as 3+ by immunohistochemistry or HER2 amplification by fluorescent *in situ* hybridization (HER2/CEP17 >2.0).

### Endpoints

The primary endpoint was the response rate. Tumor shrinkage was evaluated according to the RECIST version 1.0 (6), and response was categorized as CR, PR, SD or PD. Bone lesions are generally considered non-target lesions as they are unmeasurable. However, bone is a common site of metastasis from breast cancer, in which the rate of metastasis is as high as 70–80%. In the present study, bone metastases were considered target lesions for the evaluation of response only in patients who only had bone metastases. The response of bone lesions was evaluated according to the standards of the Japanese Breast Cancer Society (7). If lesions existed in sites other than bone, bone lesions were evaluated as non-target lesions.

Secondary endpoints were PFS, OS, CBR and safety. PFS was defined as the number of days from enrollment to an initial event (disease progression or mortality from any cause, whichever occurred first). CBR was defined as the percentage of patients who had a CR, PR or prolonged SD maintained for at least 24 weeks among all eligible subjects. Safety was evaluated according to the Common Terminology Criteria of Adverse Events (CTCAE), version 3.0 (8).

### Statistical analysis

The design of this study was based on a binomial distribution with no planned interim analysis. Assuming a null hypothesis of a 6% ORR and an alternative hypothesis of a 20% ORR, with one-sided type I error = 0.025 and type II error = 0.2, the required sample size was calculated to be 33. The planned sample size was set at 35, with the consideration of ~5% of patients being ineligible.

Exact confidence intervals (95% CI) were calculated for CBR and ORR. PFS and OS were estimated by the Kaplan-Meier method. The incidence of grade 3 or 4 adverse events is shown according to type. If an adverse event of the same type and the same grade developed twice in the same patient, it was counted as one event. Statistical analysis was performed with SAS System Release 9.1.3 (SAS Institute Inc., Cary, NC, USA).

## Results

### Patient characteristics

From September 2008 to November 2010, a total of 37 patients were enrolled in the study. The patients were followed up and outcomes were confirmed in February 2012. Table I shows the demographic characteristics of the 37 patients. The median age was 43.0 years (range, 33–53), and the median body-mass index was 21.6 kg/m^2^ (range, 16.9–30.3). The median disease-free interval (DFI) was 58.0 months (range, 0.9–201.3) and 12 patients (42.9%) had longer DFI (>60 months). ER/PgR status was ER+/PgR+ in 27 patients (73.0%), ER+/PgR− in 8 (21.6%) and ER−/PgR+ in 2 (5.4%). HER2 was negative in 36 patients (97.6%). During prior treatment with an LH-RH analogue plus TAM, 26 patients (70.3%) had PD, and 6 (16.2%) had recurrence during postoperative adjuvant therapy; 5 patients (13.5%) had completed the previous course of adjuvant therapy. Previous treatment included radiotherapy in 13 patients (35.1%) and chemotherapy in 20 (54.1%).

Thirty-one patients had distant metastases and 6 had locally advanced disease. The sites of metastasis were bone in 14 patients, lymph nodes in 12, liver in 9, lung in 9, contralateral breast in 2, distant skin in 2 and pleura in 1. Thirty-six patients (97.3%) had measurable disease, 21 (56.8%) of the patients also had bone lesions and 1 had only bone metastasis.

### Clinical effectiveness

**Figure 1:**
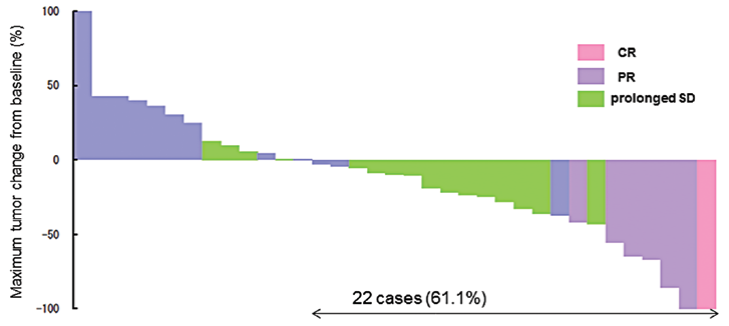
Waterfall plot of maximal change (%) in RECIST-evaluable tumor size from baseline. Thirty-six patients had measurable disease at baseline, and tumor shrinkage was found in 22 patients (61.1%). Of the patients with long-SD, 12 patients (75%) had tumor shrinkage. CR, complete response; PR, partial response; SD, stable disease.

Clinical effectiveness is summarized in Table II. One patient (2.7%) had a CR, and 6 (16.2%) had PR for a response rate of 18.9% (95% CI, 8.0% to 35.2%; P=0.006 under the null hypothesis of a 6% ORR). Sixteen patients (43.2%) had prolonged SD. The CBR was thus 62.2% (23 patients, 95% CI, 44.8–77.5%). Eleven patients (29.7%) had PD. One patient with a response of not evaluable withdrew her informed consent as she wanted to receive a folk remedy. Fig. 1 shows a waterfall plot of maximal change (%) in RECIST-evaluable tumor size from baseline. Thirty-six patients had measurable disease at baseline, and tumor shrinkage was found in 22 patients (61.1%). Of the patients with prolonged SD, 12 patients (75%) had tumor shrinkage.

Regarding the previous treatment (LH-RH analogue + TAM) status, the ORR of the patients was as follows; 16.7% (1/6) in the recurrence group during postoperative therapy, none (0/1) in the recurrence group within 1 year after completing postoperative therapy, none (0/4) in the recurrence group during continued adjuvant therapy with TAM alone or within 1 year after completion, and 23.1% (6/26) in the disease progression group during treatment for advanced or recurrent breast cancer.

### Patient outcomes

**Figure 2:**
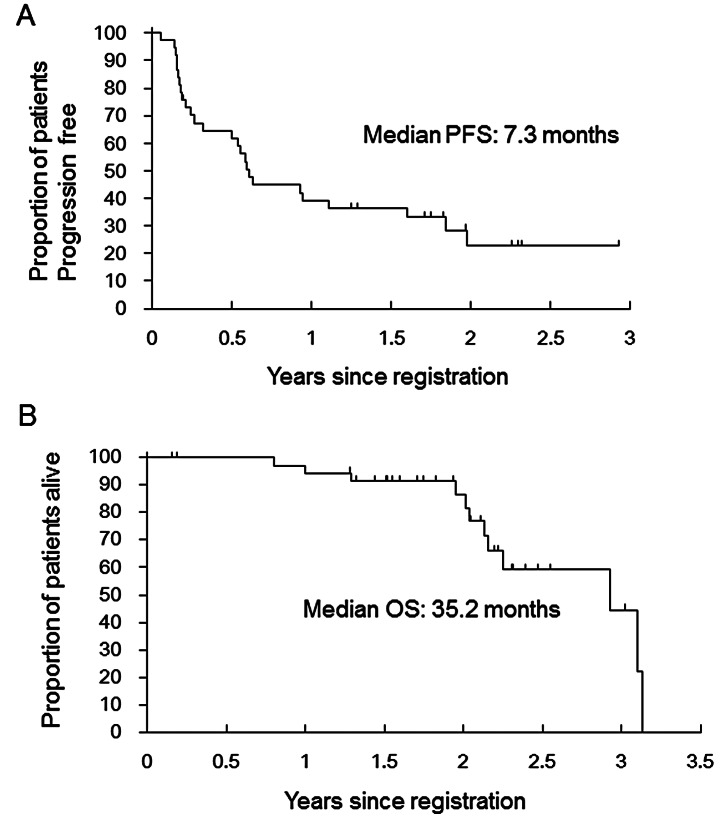
(A) Progression-free survival (PFS) and (B) overall survival (OS) since registration of the 37 enrolled patients. The median PFS and OS were 7.3 and 35.2 months, respectively. New lesions developed in 12 patients, 9 had progression of non-target lesions and 1 had progression of target lesions. Breast cancer was responsible for the 12 deaths.

Fig. 2 shows PFS and OS. The median PFS was 7.3 months. New lesions developed in 12 patients, 9 had progression of non-target lesions, and 16 had progression of target lesions. The median OS was 35.2 months. Breast cancer was responsible for the 12 deaths.

### Adverse events

Adverse events are shown in Table III. Most adverse events were grade 1. One patient had grade 2 arthralgia and 1 had a grade 2 bone fracture. Adverse drug reactions for which a causal relationship to treatment could not be ruled out are shown. A total of 13 events occurred in 8 patients. With the exception of the grade 2 arthralgia (1 patient), all other events were grade 1. Treatment was not discontinued due to adverse events in any patient. There were no safety issues according to the IDMC.

## Discussion

Few confirmatory studies have been performed with aromatase inhibitors in combination with luteinizing hormone-releasing hormone (LH-RH) analogue in premenopausal women with recurrent or advanced breast cancer. Therefore, we studied the clinical effectiveness of creating a goserelin (GOS) and anastrozole (ANA) combination therapy for breast cancer patients who failed to respond to an LH-RH analogue plus tamoxifen (TAM). The response rate was 18.9%, with a clinical benefit rate (CBR) of 62.2%, a median progression-free survival (PFS) of 7.3 months, and a median overall survival (OS) of 35.2 months. On disease progression, second-line treatment options include other types of endocrine therapy for estrogen receptor (ER)-positive breast cancer. Moreover, hormone resistance includes primary (*de novo*) and secondary (acquired) resistance, and the mechanism of resistance between them may differ. It was reported (9) that the patients with secondary resistance responded to the second-line treatment. According to the previous treatment status (LHRH analogue + TAM), the objective response rate (ORR) in the patients (possibly primary resistance) with recurrence during adjuvant therapy or within 1 year after completion was low [total, 9.1% (1/11)]. On the other hand, the ORR was high (23.8%, 6/26) in the patients with disease progression during treatment for advanced or recurrent breast cancer. Although there were several cases with longer disease-free interval (DFI) (possibly secondary resistance), it was difficult to distinguish between primary and secondary hormone resistance in the present study.

Aromatase inhibitors have been shown to increase gonadotropin secretion and to activate ovarian function in premenopausal women (10,11). By contrast, LH-RH analogues inhibit ovarian function and create a postmenopausal hormone environment, facilitating a response to treatment with an aromatase inhibitor. The above mentioned treatment suggests that the combination of aromatase inhibitors with an LH-RH analogue could obtain a complete estrogen blockade by suppressing the ovarian function and the synthesis of peripheral estrogen. In addition, this treatment may produce substantial antitumor activity in premenopausal women (8). Forward *et al*(5) and Carlson *et al*(12) clearly described this hormonal environment.

A meta-analysis comparing an LH-RH analogue alone with an LH-RH analogue plus TAM in premenopausal women with advanced breast cancer showed that the ORR was 29.7 and 38.8%, the median PFS was 5.4 and 8.7 months, and the median OS was 2.5 and 2.9 years, respectively. Outcomes were significantly improved in patients who also received TAM (13). On the basis of these results, an LH-RH analogue plus TAM is currently the standard therapy for premenopausal breast cancer. Regarding the treatment of postmenopausal women with recurrent breast cancer, aromatase inhibitors can be considered a standard endocrine therapy as first-line and second-line treatments (14–18). Aromatase inhibitors appear to be a viable treatment option in combination with an LH-RH analogue given to induce a postmenopausal hormonal environment for premenopausal women with breast cancer.

In the present study, an LH-RH analogue plus an aromatase inhibitor were administered to premenopausal women who failed to respond to an LH-RH analogue plus TAM. In a separate study of first-line treatment with an LH-RH analogue and an aromatase inhibitor in 32 premenopausal women with metastatic breast cancer (12), 1 patient (3.1%) had complete response (CR) and 11 (34.4%) had partial response (PR). All patients had a clinical benefit rate (CBR) of 71.9% and a time to progression of 8.3 months (range, 2.1–63). These results were better than those obtained in our study. The majority of the patients were hormone-naïve (12), while all patients in our study were treated with an LH-RH analogue plus TAM, including the patients who developed recurrence within 1 year after the completion of postoperative treatment with an LH-RH analogue plus TAM. This data supports the recommendations of the NCCN which indicates that the patients who received prior endocrine therapy within 1 year are potential candidates for this treatment.

With regard to the second-line treatment, a retrospective study of GOS plus letrozole (n =16) in premenopausal women with advanced breast cancer (19) reported an ORR of 12.5% (1/16) and a CBR of 56.3% (9/16), which is similar to the results obtained in our study. Furthermore, our prospective study demonstrates the benefits of the GOS plus ANA treatment in premenopausal women refractory to an LH-RH analogue with TAM.

The Austrian Breast and Colorectal Cancer Study Group trial 12 (ABCSG-12) compared an LH-RH analogue plus TAM with an LH-RH analogue plus an aromatase inhibitor as an adjuvant therapy in premenopausal women with endocrine-responsive breast cancer (20). They found that there was no significant difference between the two endocrine therapy groups and that further observation is necessary. In a retrospective study evaluating the effectiveness of letrozole plus an LH-RH analogue administered concurrently with preoperative chemotherapy and as an adjuvant treatment in premenopausal women with locally advanced ER-positive breast cancer (21), the pathological CR rate, decrease in Ki-67 level, and a higher 5-year disease-free survival rate were significantly improved compared to those in a control group of similar patients who received preoperative chemotherapy followed by TAM plus and an LH-RH analogue after surgery.

The STAGE study by Masuda *et al*(22) was a randomized, double-blind trial of ANA vs. TAM in patients receiving GOS for premenopausal breast cancer in the neoadjuvant setting. The study showed that ANA demonstrated a superior benefit-risk profile compared with TAM as a neoadjuvant treatment in premenopausal women with ER+ breast cancer receiving GOS.

Only 1 patient in our study had a grade 2 adverse drug reaction (arthralgia) and the rest had grade 1 events. No patient discontinued treatment due to adverse events, which were relatively low and were considered symptoms associated with ANA in postmenopausal women. Previous studies have also reported that GOS plus ANA is safe, with no serious adverse events (12).

In conclusion, our results suggest that combination therapy with GOS and ANA is a safe, highly effective, viable treatment for premenopausal women with hormone-sensitive, recurrent or advanced breast cancer. We consider that GOS plus ANA will be recognized as a standard treatment for premenopausal ER-positive recurrent breast cancer, particularly when TAM is contraindicated or ineffective. Further studies and discussion are required to support these results.

## Figures and Tables

**Table I:** Patient characteristics.

Characteristics (n=37)	Median	Range
Age (years)	43.0	33–53
BMI (kg/m^2^)	21.6	16.9–30.3
Disease-free interval (months; 28 recurrent cases)	58.0	0.9–201.3

Characteristics (n=37)	No. of patients	%

ER and PgR status		
ER+ and PgR+	27	73.0
ER+ and PgR−	8	21.6
ER− and PgR+	2	5.4
HER2 status		
Negative	36	97.3
Unknown	1	2.7
Description of previous treatment (LH-RHa + TAM)		
Recurrence during postoperative therapy	6	16.2
Recurrence within 1 year after completing postoperative therapy	1	2.7
Recurrence during continued adjuvant therapy with TAM alone or within 1 year after completion	4	10.8
Disease progression during treatment for advanced or recurrent breast cancer	26	70.3
History of other previous treatments		
Prior radiotherapy	13	35.1
Prior chemotherapy	20	54.1
Presence of metastatic sites (n=37)		
No	6	16.2
Yes	31	83.8
Metastatic sites (n=31)		
Breast	2	6.5
Skin	2	6.5
Lymph nodes	12	38.7
Bone	14	45.2
Lung	9	29.0
Pleura	1	3.2
Liver	9	29.0
Type of treated lesions (n=37)		
Measurable disease	15	40.5
Measurable + bone	21	56.8
Bone only	1	2.7

LH-RHa, luteinizing hormone-releasing hormone analogue; TAM, tamoxifen; HER2, human epidermal growth factor receptor 2; ER, estrogen receptor; PgR, progesterone receptor.

**Table II:** Objective response rates and clinical benefit rates.

Response	No. of patients	%	95% CI
Complete response	1	2.7	
Partial response	6	16.2	
Objective response	7	18.9	8.0–35.2
Stable disease ≥24 weeks	16	43.2	
Clinical benefit	23	62.2	44.8–77.5
Stable disease <24 weeks	2	5.4	
Progressive disease	11	29.7	
Not evaluable^a^	1	2.7	

a Response was not assessable in 1 patient who withdrew her informed consent as she wanted to receive a folk remedy.

CI, confidence interval.

**Table III:** Adverse events and adverse drug reactions.

	Adverse events		Adverse drug reactions	
		
Event	Grade 1	Grade 2	Grade 1	Grade 2
Hot flashes	9		3	
Joint pain	5	1	1	1
Sweating	7		1	
Laboratory abnormalities^a^	3		3	
Insomnia	3		1	
Pain (limbs)	3			
Arthritis (non-septic)	2			
Fracture^b^		1		
Precordial pain	1		1	
Fatigue	1		1	
Nausea	1		1	

a Laboratory abnormalities: abnormal RBC, total cholesterol and ALT values occurred in 1 patient each.

b Fracture: a fissured fracture occurred after stumbling. There were no grade 3 or 4 adverse events.
